# Association of multi-criteria derived air toxics hazard score with lung cancer incidence in a major metropolitan area

**DOI:** 10.3389/fpubh.2023.1002597

**Published:** 2023-06-26

**Authors:** Angela Y. Zhu, Tara L. McWilliams, Thomas P. McKeon, Anil Vachani, Trevor M. Penning, Wei-Ting Hwang

**Affiliations:** ^1^Department of Biostatistics, Epidemiology, and Informatics, Perelman School of Medicine, University of Pennsylvania, Philadelphia, PA, United States; ^2^Center for Clinical Epidemiology and Biostatistics, Perelman School of Medicine, University of Pennsylvania, Philadelphia, PA, United States; ^3^Center of Excellence in Environmental Toxicology, Perelman School of Medicine, University of Pennsylvania, Philadelphia, PA, United States; ^4^Department of Geography, Temple University, Philadelphia, PA, United States; ^5^Abramson Cancer Center, Perelman School of Medicine, University of Pennsylvania, Philadelphia, PA, United States; ^6^Department of Medicine, Pulmonary, Allergy, and Critical Care Division, Hospital of University of Pennsylvania, Philadelphia, PA, United States; ^7^Department of Systems Pharmacology and Translational Therapeutics, Perelman School of Medicine, University of Pennsylvania, Philadelphia, PA, United States

**Keywords:** lung cancer incidence, environmental exposures, carcinogens, hazard score, spatial regression

## Abstract

**Background:**

Lung cancer remains a major health problem world-wide. Environmental exposure to lung cancer carcinogens can affect lung cancer incidence. We investigated the association between lung cancer incidence and an air toxics hazard score of environmental carcinogen exposures derived previously under the exposome concept.

**Methods:**

Lung cancer cases diagnosed in Philadelphia and the surrounding counties between 2008 and 2017 were identified from the Pennsylvania Cancer Registry. Age-adjusted incidence rates at the ZIP code level were calculated based on the residential address at diagnosis. The air toxics hazard score, an aggregate measure for lung cancer carcinogen exposures, was derived using the criteria of toxicity, persistence, and occurrence. Areas with high incidence or hazard score were identified. Spatial autoregressive models were fitted to evaluate the association, with and without adjusting for confounders. Stratified analysis by smoking prevalence was performed to examine potential interactions.

**Results:**

We observed significantly higher age-adjusted incidence rates in ZIP codes that had higher air toxics hazard score values after controlling for demographic variables, smoking prevalence, and proximity to major highways. Analyzes stratified by smoking prevalence suggested that exposure to environmental lung carcinogens had a larger effect on cancer incidence in locations with higher smoking prevalence.

**Conclusion:**

The positive association between the multi-criteria derived air toxics hazard score and lung cancer incidence provides the initial evidence to validate the hazard score as an aggregate measure of carcinogenic exposures in the environment. The hazard score can be used to supplement the existing risk factors in identifying high risk individuals. Communities with higher incidence/hazard score may benefit from greater awareness of lung cancer risk factors and targeted screening programs.

## 1. Introduction

Lung cancer results in the greatest number of deaths from cancer with the National Cancer Institute’s Surveillance, Epidemiology, and End Results Program (SEER) and American Cancer Society estimating 127,070 deaths due to lung cancer in 2023 (1, 2). Further, the estimated number of new cases in 2023 is expected to be 238,340, suggesting that lung cancer remains an ongoing major health problem (2). The age-adjusted incidence for cancer of the lung has been reported to be 62 per 100,000 in Pennsylvania (PA) in 2022, which is greater than the national incidence rate of 57 (3). This incidence rate is even higher in the two counties with major cities in PA, namely Philadelphia county (70.6 per 100,000) and Allegheny county (64.6 per 100,000), which contains Pittsburgh (4, 5) based on data from 2015–2019. The five-year survival, defined to be the percent of subjects who are alive five years after a lung cancer diagnosis, is 27% for Pennsylvanians based on data from 2016–2021. This percentage is slightly but significantly higher than the national five-year survival of 25% (3, 6). Research to advance lung cancer survival including improvements in screening, diagnosis, and treatment are still in urgent need.

Tobacco use through cigarette smoking is a principal risk factor for lung cancer. The cancer risk associated with smoking focuses on the chemical constituents of smoke and the mechanisms through which they may lead to cancer development (7). Potentially carcinogenic compounds that are found in tobacco smoke such as polycyclic aromatic hydrocarbons (PAHs), aromatic amines, benzene, vinyl chloride, butadiene, arsenic, and cadmium are also found in the environment as components of air pollution. Air pollutants also include components of diesel fuel combustion, e.g., nitro-arenes which have cancer causing potential (8–10). These exposures may contribute to lung cancer incidence and deaths in people who have never smoked–up to 20% of lung cancer deaths in the United States occur in never smokers (11). The role of outdoor air pollution in cancer risk is further supported by the International Agency for Research on Cancer (IARC)‘s designation of air pollution as a Group 1 carcinogen as an agent known to be carcinogenic to humans (12). Air pollution has also been shown to be a lung tumor promoter where exposure to PM2.5 leading to inflammation is the culprit (13). However, few studies have considered carcinogen emissions in their totality.

Inspiring by the exposome concept proposed by Christopher Wild in 2005 (14), which considers all exposures to an individual in his or her lifetime and relates them to health outcomes, McKeon et al. proposed a methodology to construct a hazard index to measure the combined effects of chemical compounds that may lead to higher lung cancer incidence (15). Using a Multi-Step Multi-Criteria Decision Analysis (MMCDA) risk assessment framework, the air toxics hazard index is designed to summarize the relative impact of many chemicals in a particular study area using a point system. This point system quantifies chemicals in terms of their toxicity based on IARC classifications and literature-based evidence that they possess the characteristics of chemical carcinogens, persistence as indicated by status as a volatile organic compound (VOC), and occurrence in terms of the amount and frequency of emission in the study area. In their report, they utilized more than 30 years of data from US EPA’s Toxic Release Inventory (TRI) for chemicals that potentially cause lung cancer beyond the ones found in air pollution and computed the hazard index for Philadelphia and surrounding counties. Although it is understandable that their proposed air toxics hazard index still does not fully capture the complexity of the exposome in its strict definition, the consideration of multiple criteria including toxicity (based on characteristics of carcinogens that are agnostic and independent of organ site), persistence, and occurrence which has both a temporal and geospatial component still provides a means for mirroring many of the key concepts of exposome components.

The objective of this study was to investigate the association between lung cancer incidence and this hazard index now referred to as air toxics hazard score to prevent confusion with a classical definition of hazard index. Using cases derived from the Pennsylvania Cancer Registry, we conducted ZIP code level spatial regression analyzes to examine this association for Philadelphia and surrounding counties while controlling for relevant demographics and other covariates including smoking prevalence. Geospatial analyzes including the use of maps and various cluster detection and regression techniques are useful to investigate geographic patterns in a health-related outcome such as cancer incidence and explore risk factors including socio-, behavioral or environmental factors (16–18). A significant association between lung cancer incidence and the air toxics hazard score would support the validity of this hazard score as an aggregate measure of carcinogenic exposures in the environment and the use of the air toxics hazard score as a risk stratification tool to supplement the existing risk factors in identifying high-risk individuals.

## 2. Methods

### 2.1. Data sources

Our study area consisted of 212 ZIP codes in five counties located in southeastern Pennsylvania: Bucks, Chester, Delaware, Montgomery, and Philadelphia, which comprise the major metropolitan area of Philadelphia and the surrounding suburbs. Such geographic area is typically used for similar research studies because these areas are highly susceptible to toxic environmental exposure due to considerable human activities and often with higher cancer incidence because of the large population size. Case data were identified to include all patients diagnosed with lung and bronchus cancer between 2008 and 2017 who resided in the five above-mentioned counties at the time of diagnosis using the Pennsylvania Cancer Registry using the following ICD 10 diagnosis codes: C340 (main bronchus), C341 (upper lobe, bronchus or lung), C342 (middle lobe, bronchus or lung), C343 (lower lobe, bronchus or lung), C348 (overlapping sites of bronchus and lung), and C349 (unspecified part of bronchus or lung). Cases were excluded if they involved *in situ* and non-carcinoma histology, were not uniquely matched with a census tract ID, or their age at diagnosis belonged to an age group with zero population size as estimated by US Census Bureau indicating a possible data error for the registry. Complete details regarding the selection of the study population and determination of geographical location were previously published in Zhu et al. (19). We used 10-year incident rates to avoid the noise from the yearly variations in the incidence rates and to increase the statistical precision for the incidence rate estimates by including more cases. The final sample size for analysis was comprised of 30,165 cases.

The population size for each ZIP code was obtained from the US Census Bureau (20). We chose to use ZIP code as the geographical unit of interest in the current analyzes because of its familiarity in communications with health care providers and the general population. Although there are merits of using other geographical units such as census tract, we believe proper covariate adjustment can remove some sensitivity of ZIP code level analysis due to varying population size. Many published reports have also demonstrated that findings from ZIP code level analysis have similar utility to those that used census tracts as the unit of analysis (21–23).

For demographic covariates, median age, percentages of male, white race, Hispanic ethnicity, high school education or less, below poverty level, median household income, and population density in each ZIP code were obtained using 2010–2014 American Community Survey (ACS) 5-year estimates. The 2010–2014 ACS survey data were selected because the years coincided with the halfway point of the study period. The distance to highway was calculated in ESRI’s *ArcMap* to represent the number of meters from the centroid of a ZIP code to the nearest Class 1 or Class 2 highway as shown in the PA ZIP codes and the major highways shape files (24, 25). Estimates of smoking prevalence at the ZIP code level were obtained from the Centers for Disease Control and Prevention’s (CDC) PLACES 2020 release as derived from the 2017/2018 Behavioral Risk Factor Surveillance System (BRFSS) survey (26, 27). Data from 2017/2018 were used because ZIP code level smoking prevalence from earlier years were not available for our study area. While there is a slow decline in smoking prevalence observed over the years in Pennsylvania (28, 29), it was reasonable to assume that the relationship between smoking and lung cancer incidence remained consistent over the study period and the longitudinal patterns in smoking prevalence were similar across the ZIP codes. Data from the U.S. Environmental Protection Agency (EPA)‘s Toxic Release Inventory (TRI) program, which collects information on the management and emission of toxic chemicals into the environment, were obtained from EPA’s Data Mart website (30).

### 2.2. Air toxics hazard scores

For each ZIP code, we calculate the air toxics hazard score using the MMCDA approach as described previously in McKeon et al. based on TRI data between 1987 and 2007 (15). We choose 1987 because it is the first year for which TRI data became available, and the year 2007 is chosen to maintain the temporal order of the exposures and the years for which the lung cancer cases were included (29). Furthermore, using a wider time frame would also avoid the results to be sensitive to the uncertainty regarding the length of the latency period. Multi-criteria decision analysis (MCDA) is an established framework for several decades to guide decision that requires consideration of multiple domains (31). MCDA has been implemented in many application areas and is widely used by US EPA for different investigations including exposure research (32–34) and risk assessment (35, 36). In our previous work (15), we developed the air toxics hazard score by modifying the MCDA that was originally applied to hydraulic fracturing fluids (37, 38) to consider the domains of toxicity, persistence, and release amounts in which the release amounts was used as a weighting domain. TRI records the amounts of chemical emissions (in pounds) released into the air (both fugitive and stack emissions) by industrial and federal facilities that manufacture, process, or use toxic chemicals each year with one data entry per emission per chemical per facility. Specifically, chemicals reported in TRI data are included in the current evaluation if they meet one of the following five exposome features: (i) they are classified as an IARC group 1 to 3 carcinogen, (ii) are one of the EPA 16 priority PAHs, (iii) found in diesel exhaust, (iv) are deemed a VOC by the EPA, and/or (v) are shown to contribute to lung carcinogenesis based on the literature that they possess the characteristics of chemical carcinogens (8, 12, 39–41). Although IARC classifications are in general carcinogen specific and not necessarily specific to lung cancer, the inclusion of chemicals per IARC designation aims to capture a complex mixture of man-made chemicals related to outdoor air pollution which is considered to be Group 1 carcinogen by IARC (12). We believe that it would be incorrect to dismiss the known carcinogenicity of these compounds even though they may not be lung carcinogens *per se* since it is not possible to prove a negative. These selected chemicals are then scored according to the criteria of toxicity, persistence, and occurrence with a point system. The raw scores for these chemicals are calculated based on their chemical toxicity and persistence and then rescaled so that they are between 0 and 1. A chemical’s risk score $S^{risk}$ is the sum of the rescaled toxicity and persistence scores with higher values indicating greater risk. Lastly, the occurrence score $S^{occurrence}$ considers the amount of a chemical released in a ZIP code relative to the total emission amount of that chemical in the entire study area. The final air toxics hazard score for each ZIP code *i*, $HS_{i}$ , is calculated over all the selected chemicals indexed by *j* using the equation shown below. A higher hazard score indicates higher level of exposure to the chemicals considered in the derivation.


$$HS_{i}=\underset{j}{\sum }S_{j}^{occurrence}\times S_{j}^{risk}$$

For 209 chemicals reported in TRI between 1987 and 2007, 109 of them met at least one of the five exposome features: 80 were classified as IARC group 1 to 3 carcinogen, 5 were one of the EPA 16 priority PAHs, 6 were found in diesel exhaust, 44 were deemed a VOC by the EPA, and 9 were shown to contribute to lung carcinogenesis based on the literature that they possess the characteristics of chemical carcinogens. The selected chemicals are provided in Supplementary Table S1 by McKeon et al. (15).

### 2.3. Descriptive analysis and spatial autocorrelations

Descriptive statistics including mean, standard deviation (SD), median, minimum, maximum, frequency, and percentage were computed for ZIP code level study variables. Our primary outcome was the age-adjusted lung cancer incidence in the 10-year interval, 2008–2017, and the main independent variable of interest was the air toxics hazard score based on TRI emission data from 1987 to 2007 to allow for the latency period between exposure and disease occurrence. We used age-adjusted incidence rates via the adjustment of crude incidence rates according to the 2000 U.S. Standard Million Population, which is the most recent standard population available, to account for differences in cancer incidence in different age groups. This adjustment procedure assumed a total population of 1,000,000 people and allocated the population into 13 age groups (0–4, 5–9, 10–14, 15–19, 20–24, 25–34, 35–44, 45–54, 55–59, 60–64, 65–74, 75–84, 85, and above) (42). The outcome variable, age-adjusted incidence rate, was assumed to follow a normal distribution after examining the distribution using a histogram.

We created maps for the incidence rates and hazard score values by ZIP code to assess the spatial distributions descriptively over the study region. To assess the spatial autocorrelation, a Moran’s I statistic was computed using queen contiguity spatial weight matrix such that ZIP codes sharing a side or corner are given a spatial weight of 1 and others have a spatial weight of 0 (43, 44). The significance of Moran’s I was tested with a permutation test. A positive Moran’s I (i.e., positive spatial autocorrelation) would indicate that the observed values of the same variable (e.g., incidence rate) from two different locations that are near one another are more similar than those that are more distant (45, 46).

Because almost half of the ZIP codes had a hazard score of 0, we created a 3-level categorical hazard score groups by applying the Jenks natural breaks algorithm for the ZIP codes with hazard score greater than 0. The Jenks natural breaks algorithm is a commonly used approach to obtain the best arrangement of values into several categories such that the variance within categories is minimized and the variance between categories is maximized (47). The frequencies for the three hazard score categories were: Category “0” with 103 ZIP codes (48.6%), Category “low” with 89 ZIP codes (41.5%) for positive hazard score up to 1, and Category “high” with 21 ZIP codes (9.9%) for hazard score above 1. The correlations between the age-adjusted incidence rates, the hazard score as both the continuous and categorical versions, and the demographic variables were computed using Pearson’s correlation coefficient. Boxplots of the age-adjusted incidence rates by the 3-level hazard score groups were created and tested for any between-group difference using one-way ANOVA, followed by a Jonckheere-Terpstra (JT) test to test for trend (48).

### 2.4. Spatial autoregressive regression models

To test the central hypothesis that our proposed air toxics hazard score are associated with lung cancer incidence rates, we considered three spatial autoregressive regression models: spatial error model (SEM), spatial lag X (SLX) model, and spatial Durbin error model (SDEM) (49, 50). These three models reflect different assumptions about the relationship between independent variables *X* and outcome *Y* and about the way *X* and spatial neighbors of *Y* affect *Y*. The SEM model is given by y=Xβ+u,u=λWu+e , where $e\sim \left(0,\sigma^{2}I\right)$ with X is the set of covariates, β is the associated regression coefficient vector, u is a vector of spatial random effects, W is the queen contiguity matrix, and λ represents the average extent of spatial correlation among the errors. Next, the SLX model, given by y=WXθ+Xβ+e , where 𝑒~𝑁(0, 𝜎^2^𝐼), includes 𝑊*X* as a matrix of spatially lagged independent variables and θ as a vector of lagged effect estimates that account for the effects of X from neighboring locations. The SDEM model combines features of SEM and SLX such that y=WXθ+Xβ+u,u=λWu+e with e~N(0,I) . These models are classified as local models because they do not allow endogenous feedback effects (i.e., events in one location lead to a reaction in its neighbors; that reaction, in turn, produces a feedback response in the original location as well as other nearby locations) (51). We fitted these local models in the current study instead of the alternative global models because we expect effects of exposure to be restricted to affecting incidence rates in surrounding areas with relatively local spatial autocorrelation as is often appropriate for most applications. Conditional autoregressive model (CAR), another commonly used approach for spatially distributed data, was not employed here because it often produces results similar to the spatial autoregressive regression models.

After including the 3-level hazard score categorical variable as the main independent variable in the model, the model building process proceeded in a stepwise fashion by adding or removing covariates (demographics variables, smoking prevalence, and distance to highway) that were correlated with the incidence rate at the 0.1 significance level in the univariate analysis one at a time. Inclusion is based on AIC, and the process continues until the model reaches the smallest AIC. For the variable of distance to highway, we observed the functional form of a linear spline at knot = 5 km to have the best fit with the observed pattern after examining the residual plots from a model that included it as a continuous variable. We only considered the categorical version of the hazard score for the regression analysis because the skewness of the distribution and for the ease of the interpretation in the regression coefficients. The collinearity of the fitted models was examined using variance inflation factors. The final multivariable SEM, SLX, and SDEM models were derived separately and compared using the likelihood ratio and Lagrange multiplier tests (52). We only presented the results from the final SEM model because it has a significantly better fit than the SDEM and SLX models (all comparisons had *p*-values<0.05) as well as the largest R-squared value and smallest AIC value. The value and the significance of Moran’s I statistic for the presented model were computed using the residuals of the corresponding model. The *lagsarlm* and *errorsarlm* functions in the *sp*. package in R were used to fit the spatial models (44).

### 2.5. Stratified analysis by smoking prevalence

To further examine the role of smoking, we performed a stratified analysis by which two spatial SEM regression models were fit separately for ZIP codes with low smoking prevalence and those with high smoking prevalence; the levels were split at the median of 16% which is similar to the 2019 national average of 14%. Only covariates selected in the final SEM model were included.

All analyzes were conducted in R version 4.1.2.

## 3. Results

### 3.1. Sample characteristics

Characteristics of 212 ZIP codes in the study areas are presented in Table 1. Approximately half of the ZIP codes had a median age of 40 years old, but the range was wide. Although half of the ZIP codes contained a population that was composed of White and non-Hispanic individuals, had over 60% of residents with more than a high school education, contained a small percentage below the poverty line, and had a median annual household income of USD 34,900, the values across ZIP codes varied significantly. Half of the ZIP codes had a smoking prevalence above 16.2%, but this prevalence ranged from 4.5 to 29.6% across the area of interest.

**Table 1:** Characteristics of 212 ZIP codes in the study area.

	Overall (*N* = 212)	
	Mean (SD)	Median [Min, Max]
Median age (years)	40.0 (7.31)	40.5 [20.2, 83.4]
Female sex (%)	51.1 (6.71)	51.4 [0, 86.5]
White race (%)	77.4 (25.0)	87.7 [2.30, 100]
Hispanic (%)	5.85 (8.85)	3.30 [0, 65.3]
High school education or less (%)	39.5 (17.6)	39.9 [6.30, 100]
Poverty (%)	10.5 (10.5)	6.45 [0, 57.8]
Median income (10,000 USD)	3.49 (1.42)	3.37 [0.406, 16.3]
Population density (10 people per sq. mile)	501 (630)	230 [4.22, 3,360]
Smoking prevalence (%)	16.7 (4.72)	16.2 [4.50, 29.6]
Smoking categories		
High	111 (52.4%)	
Low	101 (47.6%)	
Distance to major highway (km)	2.08 (2.19)	1.25 [0.00509, 10.5]

### 3.2. Mapping of air toxics hazard score and age-adjusted lung cancer incidence

**Figure 1:**
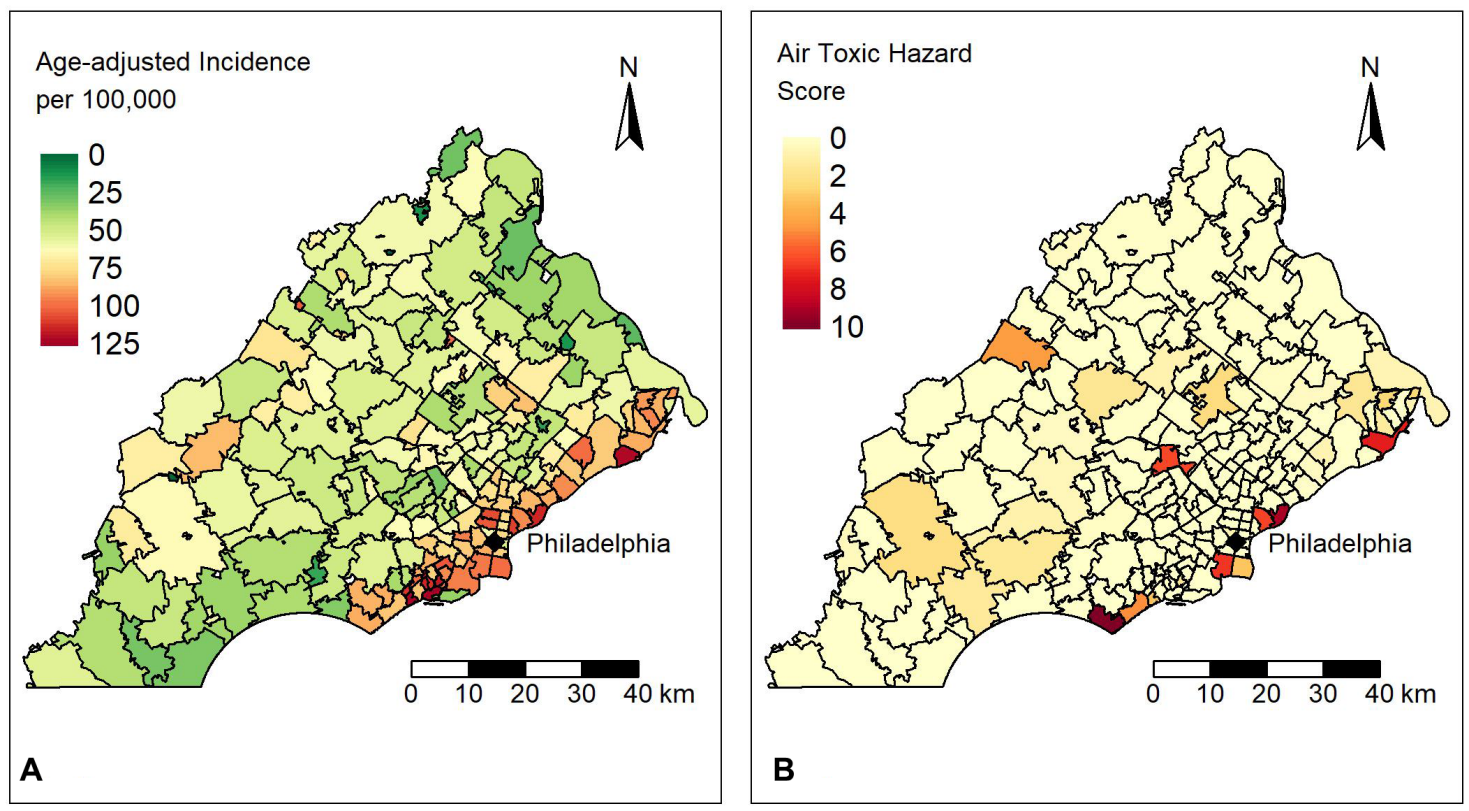
**(A)** Distribution of age-adjusted lung cancer incidence rates per 100,000 people for ZIP codes in the study area. **(B)** Distribution of the air toxics hazard scores for ZIP codes in the study area.

The mean and median age-adjusted lung cancer incidence rate across 212 ZIP codes were 62.1 (SD 24.5) and 60.4 (range 125) per 100,000, respectively. The mean air toxics hazard score was 0.42 for the study area while the median was close to zero, suggesting that most areas have relatively low exposure, but there are certain locations with much larger hazard score values (range 9.63). Figure 1 shows (a) the age-adjusted incidence rates for lung cancer and (b) the air toxics hazard score for lung cancer in the study area. We observed that ZIP codes with high incidence rates lie along the southeastern border of our study area. In some instances, the incidence rate was twice as high as the mean or median values. The Moran’s I statistics for the incidence rates and hazard score were 0.41 and 0.13 (all *p*-values<0.001), respectively, which suggested a significant positive spatial autocorrelation, so that spatial autocorrelation regression models may be required.

### 3.3. Association between air toxics hazard score and age-adjusted incidences

**Figure 2:**
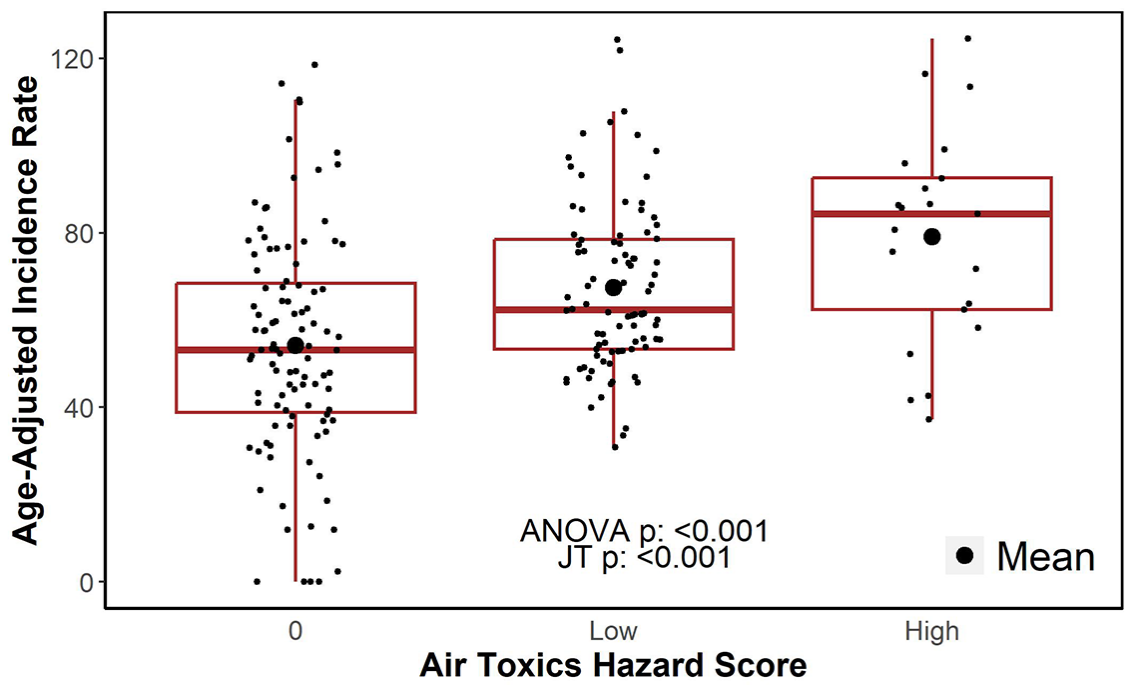
Age-adjusted incidence rates by air toxics hazard score category. JT: Jonckheere-Terpstra test for trend.

A boxplot of age-adjusted incidences by the categories of the hazard score indicated a positive relationship as shown in Figure 2. The difference in the age-adjusted incidences between hazard score groups was statistically different from zero (one-way ANOVA, value of *p*<0.001). Specifically, the average differences in incidence between the ZIP codes in the low and high hazard score categories versus that of the ZIP codes in the zero (0) hazard score group (i.e., reference category) was estimated to be 13.19 (95% CI: 6.58–19.80) and 24.93 (95% CI: 14.02–35.84) per 100,000, respectively. The increase in the age-adjusted incidence rates as the hazard score increases suggested a significant dose–response relationship (*JT test* for trend, value of *p*<0.001). That is, the incidence of lung cancer appeared to be larger for ZIP codes that have greater exposure to toxic chemicals.

### 3.4. Spatial autocorrelation regression of the multivariable model

Table 2 shows the correlations between the age-adjusted incidence rates, air toxics hazard score, and demographic characteristics. We observed significant correlations between the age-adjusted incidence rates with both the continuous and the categorical hazard score with a correlation coefficient of 0.253 and 0.344, respectively (all value of ps<0.01). Age-adjusted incidence rates were also significantly correlated (all value of *p*<0.05) with all demographic variables examined except for sex with the magnitude of the correlation coefficients ranging from 0.554 for smoking prevalence to 0.127 for the percentages of Hispanics.

**Table 2:** Correlations between age-adjusted incidence rates, air toxics hazard scores, and demographic characteristics.

	Incidence	Air Toxics Hazard Scores	
	Overall	Continuous	Categorical
Hazard (continuous)	0.253^***^		
Hazard (categorical)	0.344^***^		
Median age (year)	−0.268^***^	−0.111	−0.177^***^
Female (%)	0.094	−0.006	−0.063
White (%)	−0.343^***^	−0.047	−0.115^*^
Hispanic (%)	0.127^*^	0.114^*^	0.145^**^
High School or less (%)	0.488^***^	0.227^***^	0.245^***^
Median income (10,000 USD)	−0.334^***^	−0.145^**^	−0.179^***^
Poverty (%)	0.436^***^	0.181^***^	0.230^***^
Population density (10 people per sq. mile)	0.390^***^	−0.014	0.001
Distance to major highway (km)	−0.343^***^	−0.145^**^	−0.209^***^
Smoking prevalence (%)	0.554^***^	0.261^***^	0.3056^***^

* *p*-value < 0.10,

** *p*-value < 0.05,

*** *p*-value < 0.01.

The statistical significance of the correlation coefficients is indicated by asterisks.

Table 3 presents the regression coefficient from the fitted SEM with and without adjusting for demographics and smoking prevalence along with the corresponding 95% CIs and *p*-values. The model specified that


$$\begin{array}{l} LungCancerIncidence=7.49hazard_{low}+11.42hazard_{high}\_\\ 0.42hispanic+0.28highschool+\\ 0.06popdensity+1.5smoking\_\\ 3.82distance_{under5km}+\\ 0.59distance_{over5km}+0.32Wu \end{array}$$

where W is a queen contiguity matrix and u is a vector of spatial random effects. Based on the SEM model, higher hazard score categories were associated with increases in age-adjusted incidence after adjusting for covariates in the model and spatial autocorrelations supports the study hypothesis that higher values of air toxics hazard score are associated with increased lung cancer incidence. ZIP codes that fall within the low and high hazard score categories were associated with an average increase of 7.5 (95% CI: 2.2–12.8) and 11.4 (95% CI: 2.4–20.5) per 100,000 in the age-adjusted incidence rates, respectively, as compared to ZIP codes with hazard score of 0. The adjusted differences were statistically different from 0 with value of ps of 0.006 and 0.013, respectively. The spatial effects coefficient, λ , was also significant with value of p of 0.004, indicating that there were significant correlations among the ZIP codes, and the use of a spatial model to account for these was warranted. Lower percentage of Hispanics individuals, higher percentage of residents with high school education or less, higher population density, higher smoking prevalence, and closer proximity to major highways within 5 km were also associated with higher incidence rates in the study area. Estimates of the main effects of smoking prevalence were significant, indicating its importance in disease risk. The values of the variance inflation factor (VIF) did not suggest issues with collinearity (all VIFs<5). Significance of the spatial autocorrelation among the residuals from the multivariable SEM model was not detected (value of *p*>0.05) based on a Moran’s I test suggesting spatial autocorrelation had been adequately captured by the final SEM model.

**Table 3:** Spatial error regression model adjusting for demographic covariates and continuous smoking prevalence.

	SEM			
	Univariate		Stepwise	
	Est (95% CI)	*P*-value	Est (95% CI)	*P*-value
Air Toxics Hazard Score				
[0]	Ref	--	Ref	--
Low, [0–1]	12.09 (6.56, 17.62)	**<0.001**	7.49 (2.19, 12.79)	**0.006**
High, [1–10]	17.89 (8.65, 27.14)	**<0.001**	11.42 (2.36, 20.47)	**0.013**
Hispanic (%)			−0.42 (−0.76, −0.08)	**0.016**
High school or less (%)			0.28 (0.01, 0.54)	**0.043**
Population density (100 ppL per sq. mile)			0.06 (−0.00, 0.11)	0.050
Smoking prevalence (%)			1.50 (0.41, 2.59)	**0.007**
Distance to major highway (km)				
≤5 km			−3.82 (−5.78, −1.86)	**<0.001**
>5 km			0.59 (−3.55, 4.72)	0.781
Lambda	0.62 (0.49, 0.75)	**<0.001**	0.32 (0.11, 0.47)	**0.004**
Model Statistics				
Log likelihood	−932.72		−907.72	
R-squared	0.350		0.487	
AIC	1875.4		1835.4	
Moran’s Index	−0.054	0.868	−0.017	0.613

Est, regression coefficient estimates; ppl, people. *P*-values < 0.05 were bolded.

### 3.5. Stratified analysis by smoking prevalence

**Figure 3:**
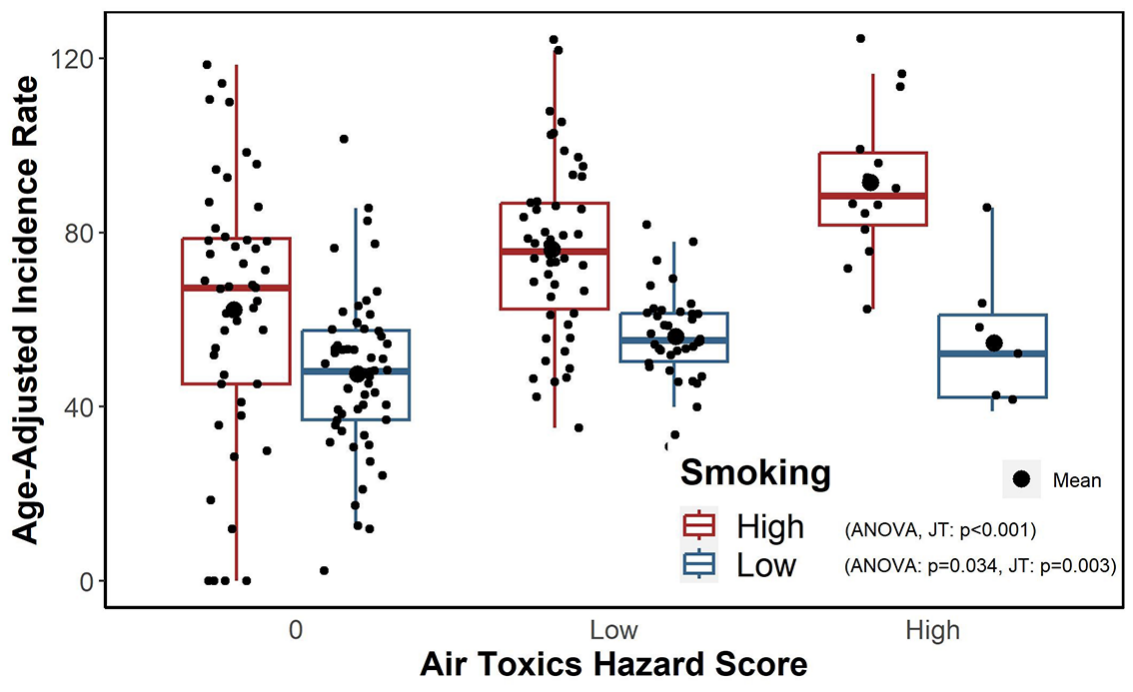
Age-adjusted incidence rates within combinations of the air toxics hazard score categories and high and low smoking prevalence levels.

For the stratified analysis by smoking prevalence levels (“high” >16% versus “low” ≤ 16%), Figure 3 indicates that the differences in incidence rates among air toxics hazard score categories were less prominent in the ZIP codes with low smoking prevalence while a much clearer dose–response pattern was observed at high smoking prevalence. Specifically, higher hazard score categories were associated with higher incidence rates in the ZIP codes with a high smoking prevalence level (one-way ANOVA, *p* < 0.001; *JT test* for trend, value of *p*<0.001); the average differences in ZIP code level incidences between low and high hazard score categories versus the incidences for ZIP codes with zero (0) hazard score were estimated to be 13.81 (95% CI: 3.79–23.83) and 29.24 (95% CI: 14.23–4.26) per 100,000, respectively. For ZIP codes with low smoking prevalence, there is a statistically significant difference and ordered trend in the incidence rates (one-way ANOVA, *p* = 0.034; *JT test* for trend, value of *p* = 0.003) but the differences were smaller; the average differences comparing low and zero hazard score groups were estimated to be 8.56 (95% CI: 1.98–15.13) per 100,000, and 7.02 (95% CI: −5.52-19.55) per 100,000 comparing high and zero hazard score groups which was not statistically significant different from 0 at 0.05 significance level given that the value 0 was contained in 95% CI.

Table 4 provides the regression estimates stratified by high and low smoking prevalence from the SEM regression after adjusting for demographic covariates and the distance to major highway variable. Estimates confirmed the observation presented in Figure 3 that even after adjusting for covariates, the hazard score categories were significantly associated with incidence rates for locations with high smoking prevalence such that ZIP codes with higher hazard score categories also had higher age-adjusted incidence rates. Similarly, the patterns for the ZIP codes with low smoking prevalence were different such that the high hazard score category compared to a hazard score of 0 was associated with an average increase in lung cancer incidence of 18.8 (95% CI: 6.1–31.5) per 100,000 (value of *p* = 0.004) for the high smoking prevalence stratum after adjusting for other covariates in the model but not for the ZIP codes with low smoking prevalence (value of *p*>0.05).

**Table 4:** Spatial error regression model estimates for low and high levels of smoking prevalence.

SEM	Low smoking (*n* = 101)		High Smoking (*n* = 111)	
	Est (95% CI)	*P*-value	Est (95% CI)	*P*-value
Air toxics hazard score				
[0]	Ref	--	Ref	--
Low, [0–1]	7.26 (1.84, 12.67)	**0.009**	7.78 (−0.37, 15.92)	0.061
High, [1–10]	1.63 (−9.44, 12.70)	0.773	18.79 (6.10, 31.49)	**0.004**
Hispanic (%)	0.29 (−0.65. 1.24)	0.545	−0.46 (−0.88, −0.05)	**0.028**
High school or less (%)	0.53 (0.29, 0.78)	**<0.001**	0.29 (−0.07, 0.65)	0.113
Population density (100 ppL per sq. mile)	0.07 (0.01, 0.13)	**0.023**	0.08 (−0.00, 0.15)	0.061
Distance to major highway (km)				
≤5 km	−2.66 (−4.60, −0.72)	**0.007**	−5.88 (−9.16, −2.59)	**<0.001**
>5 km	4.36 (0.15, 8.58)	**0.043**	−2.41 (−8.31, 3.49)	0.423
Lambda	0.24 (−0.01, 0.49)	0.110	0.30 (0.09, 0.50)	**0.015**
Model statistics				
Log likelihood	−400.84		−486.05	
R-squared	0.365		0.467	
AIC	821.7		992.1	
Moran’s index	0.014	0.363	−0.011	0.510

Est, regression coefficient estimates; ppl, people. *P*-values < 0.05 were bolded.

## 4. Discussion

In this report, we assessed the association between the age-adjusted lung cancer incidence and the air toxics hazard score, an aggregate measure of various environmental carcinogen exposures known to be related to lung cancer development, using spatial regression analysis. Using more than 30 years of data from U.S. EPA’s TRI, the air toxics hazard score for a geographic area was derived as a single value for each geographic unit that was based on the exposome concept as described by Christopher Wild (14). Although the air toxics hazard score used in the current analysis is far from perfect to fully capture the complexity and evolving definition of the exposome, we believe the score still provides a means for combining these different exposome components and can be useful in advancing the field of exposome research. The air toxics hazard scores used in the current study considered more than 200 chemicals with respect to aspects of their toxicity, persistence, and frequency of the occurrence through a MMCDA framework (15). We observed that areas with high incidence rates were concentrated in the southeastern region of Pennsylvania along its border with New Jersey, which also contained regions of high hazard scores. After adjusting for ZIP code level demographic characteristics and the distance to highway as a surrogate for traffic volume, our analyzes showed an overall pattern that reflected a dose–response relationship in that areas with higher air toxics hazard scores were associated with higher lung cancer incidence. However, this relationship differed by the smoking prevalence in the area such that a higher hazard score had a more pronounced effects in an area with high smoking prevalence. Our analysis demonstrated the value of the air toxics hazard score as a valid tool for capturing the exposome to predict lung cancer incidence.

The spatial patterns in the air toxics hazard score and lung cancer incidence suggested the importance of geographical location and the spatial autocorrelation among ZIP codes in studying lung cancer epidemiology. The significance of the spatial term in our fitted spatial regression model indicated that residuals among neighboring ZIP codes may be correlated and that our spatial approach was appropriate to account for unexplained variation of a parameter not included in the model. In addition to the use of a spatial model to control spatial autocorrelations, our findings were also strengthened by making adjustment for possible confounding by demographic variables. Our results suggested that ethnicity, education, population density, and smoking prevalence in a ZIP code are important variables that may be associated with lung cancer incidences, reinforcing findings from previous studies (53, 54). Further, an increase in distance from a major highway up to 5 km is associated with reduction in incidence, suggesting that the proximity to air pollution resulting from vehicle traffic is a risk factor. Adjusting for these covariates, ZIP codes with low and high score categories still had significantly higher incidence rates of lung cancer than those of ZIP codes with a hazard score of 0.

As smoking is considered a primary risk factor, both the adjusted analyzes and the stratified analysis in the current report provided a better understanding of its effect in the context of environmental exposures. In particular, our stratified analyzes indicated that exposure to environmental lung carcinogens tends to lead to a greater difference in lung cancer incidence in locations with a higher prevalence of smoking. It may be likely that smoking and environmental pollutants interact to further increase lung cancer risk. Although we did not investigate the mechanism of exposure in the current study, because the air toxics hazard score we used only considers carcinogenic chemical exposures for the air emissions recorded in the EPA’s TRI data, the likely exposure mechanism was through inhalation. These observations warrant consideration of both individual smoking habits and environmental exposures to lung cancer carcinogens in identifying individuals who may be at higher risk of developing lung cancer in the future. These findings also indicate a potential to use the residential address as a risk stratification tool or as a part of the eligibility criteria for lung cancer screening together with other individual (e.g., age, smoking history) and environmental risk factors (55, 56). That is, we can expand screening to include individuals living in ZIP codes with high air toxics hazard scores.

We note a few limitations of our study. Data on smoking prevalence are recent, so expected temporality could not be assessed. Our decision to utilize these data was based on the observation that changes in smoking prevalence over the years were small and would be similar across the ZIP codes. The individual smoking status was also not available. Another issue regarding temporality is that the latency period for lung cancer may be longer than what was studied based on the available TRI data. Because we used an aggregate measure of environmental exposures rather than individual chemicals or toxicants, our data provided an estimate of the combined effects of this exposome without consideration of the mode of action of this complex mixture. The current study considered newly diagnosed lung cancer cases with all stages and histology subtypes combined so that the associations of air toxics hazard score with certain stage or histology subtypes were not examined. Additionally, we used the residential address at the time of the diagnosis, and we did not capture the day-to-day movement patterns of the individuals such as travel to work or school, and the degree of the resulting exposure misclassification is unknown. Other unmeasured variables, including exposure to other environmental hazards such as radon, asbestos, and secondhand smoke, as well as a family history of or genetic predisposition to lung cancer were not available from the cancer registry, thus we cannot control them in the current analysis. Lastly, the study area was limited and, thus, the current findings may not be generalizable to other geographic areas.

Future work will consider a larger study area so that we may substantiate the relationships we observed in our current study. Moreover, a longer study period may be beneficial for capturing the temporal relationship between exposure and lung cancer development. The possible mechanisms of exposure associated with the air toxics hazard score are also warranted further investigation. The appeal of the air toxics hazard score used is that the same framework can be extended easily to a different geographical area or unit, different time frame, or even a different cancer outcome. Furthermore, because the study area covers the catchment area for Abramson Cancer Center located in Philadelphia, our goal is for the proposed framework to serve as a transferable model for other cancer catchment areas interested in better understanding exposures or risks to the populations within catchment areas. More attention should be paid to smokers who reside in areas with higher hazard scores and to non-smokers who have lived in these areas for an extended period of time. These individuals living in potentially more hazardous locations may derive the most benefit from greater uptake of public health interventions such as screening and educational programs. Furthermore, the current study also showed that locations of these high incidence and/or high air toxics hazard scores were also matched with those for environmental justice (EJ) areas (57), which are socially and economically disadvantaged and often disproportionally exposed to adverse environmental impacts and higher disease burden beyond just cancer. In recent years, PA state agencies have developed and deployed EJ Screening tools to address the needs of EJ and serve as a valuable resource for the public to better understand the potential environmental impacts of policies, projects, or health interventions. The findings of the current study provide preliminary evidence that the air toxics hazard score or the framework we used to derive and evaluate the hazard score can be part of the EJ toolbox.

Our analyzes demonstrated a positive association between the air toxics hazard score and lung cancer incidence, and the patterns of association varied by the smoking prevalence of the area. These findings support the air toxics hazard score as an useful measure for aggregating carcinogenic exposures in the environment and as a predictor of lung cancer incidence. Further validation of the air toxics hazard score are warranted. Individuals living in the communities with higher air toxics hazard scores, either with or without other risk factors, may benefit from greater awareness of lung cancer and targeted screening programs.

## Data availability statement

The data analyzed in this study is subject to the following licenses/restrictions: We conducted the present analysis under a data use agreement with the Pennsylvania Department of Health (PA-DOH). The original data from the PA-DOH Pennsylvania Cancer Registry are not available for redistribution. Requests to access these datasets should be directed to wealdinger@pa.gov.

## Ethics statement

The studies involving human participants were reviewed and approved by University of Pennsylvania Institutional Review Board (IRB number 831671). Written informed consent from the participants’ legal guardian/next of kin was not required to participate in this study in accordance with the national legislation and the institutional requirements.

## Author contributions

AZ, TP, and W-TH: study conceptualization. AZ, TLM, and W-TH: study design and statistical analysis. AZ, TLM, TPM, AV, TP, and W-TH: data acquisition and manuscript review and editing. TLM and TPM: table and figure preparation. AZ and W-TH: draft initial manuscript. All the authors read and approved the final manuscript.

## Funding

This study was supported in part by the National Institutes of Health Cancer Center Support Core Grant (P30-CA16520), and the National Institute of Environmental Health Sciences grants (P30-ES013508 and R01-ES029294 awarded to TMP). The funding sources had no involvement in conducting and reporting the study.

## Conflict of interest

AV reports personal fees as a scientific advisor to the Lung Cancer Initiative at Johnson & Johnson and grants to his institution from MagArray, Inc. and Precyte, Inc. outside of the submitted work. AV is an advisory board member of the Lungevity Foundation (unpaid).

The remaining authors declare that the research was conducted in the absence of any commercial or financial relationships that could be construed as a potential conflict of interest.

## Publisher’s note

All claims expressed in this article are solely those of the authors and do not necessarily represent those of their affiliated organizations, or those of the publisher, the editors and the reviewers. Any product that may be evaluated in this article, or claim that may be made by its manufacturer, is not guaranteed or endorsed by the publisher.
